# Supplementary material for: Integrated Metabolomics and Proteomics Dynamics of Serum Samples Reveals Dietary Zeolite Clinoptilolite Supplementation Restores Energy Balance in High Yielding Dairy Cows
Source: Metabolites. 2021 Dec 5;11(12):842. doi: 10.3390/metabo11120842 (PMC8705350; doi:10.3390/metabo11120842)

SUPPLEMENTARY INFORMATION

Figure S1. Concentrations of NEFA in control and CPL-supplementated in different time points. It is significantly lower in all samples in group 10pre-S, 5post-S and 26post-S vs. 10pre-C, 5post-C and 26post-C respectively. However, no such difference was observed in 30pre-C vs 30pre-S.

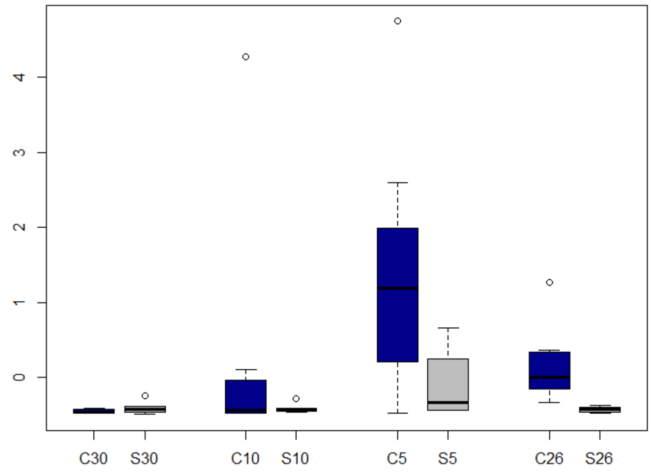

Figure S2. Concentrations of BHB in control and CPL-supplementated in different time points. BHB levels did not show significant differences between other groups which is at odds with the metabolomics data that showed decrease in BHB in all samples collected after CPL supplementation.

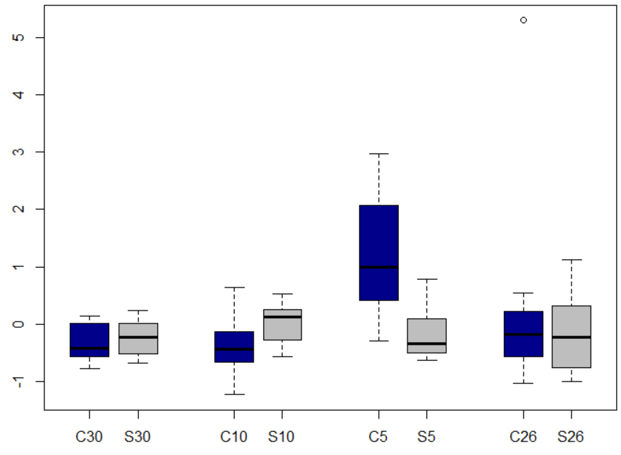

SUPPLEMENTARY INFORMATION

Figure S3. Q-Q plots of the metabolite and protein abundances were generated to assess if the abundance distributions were comparable.

METABOLITES

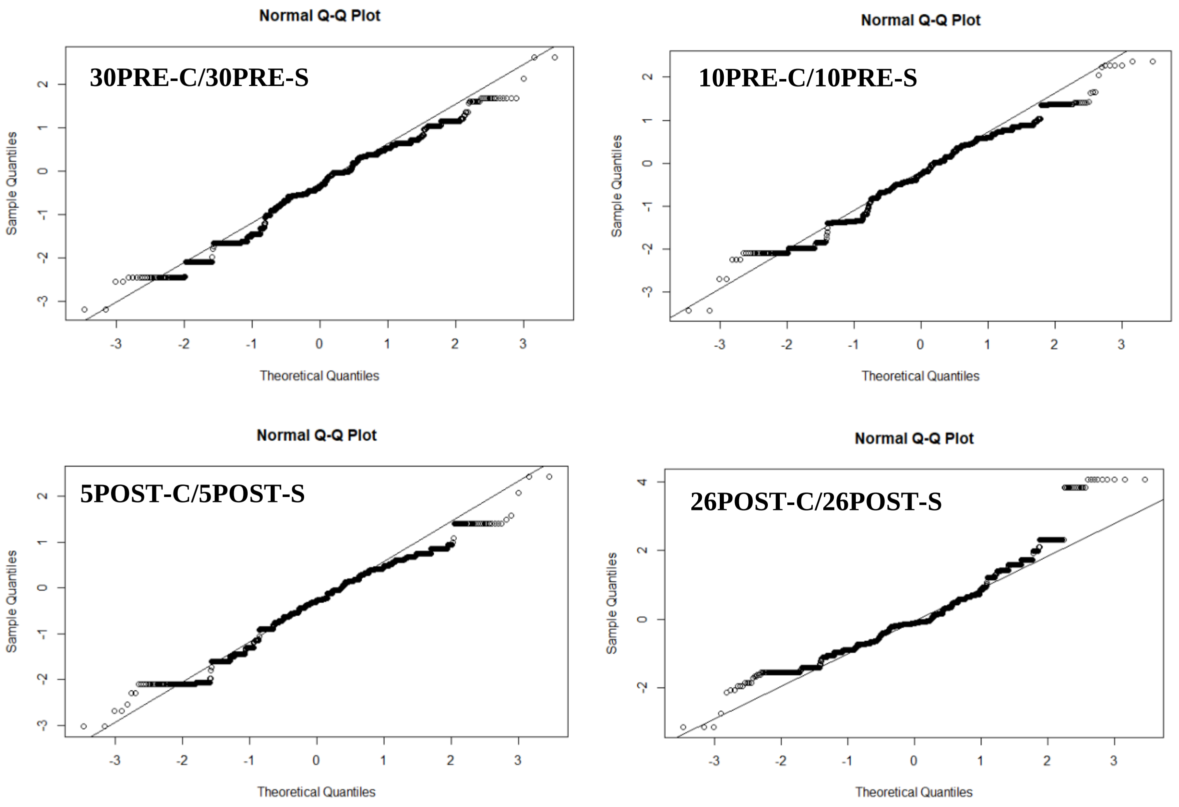

SUPPLEMENTARY INFORMATION

PROTEINS

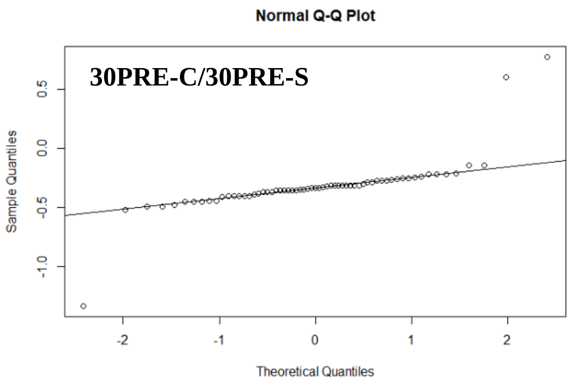

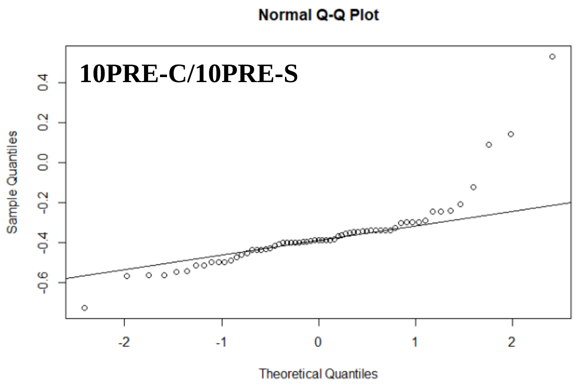

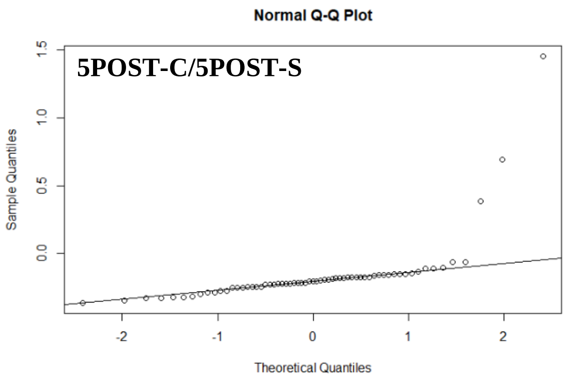

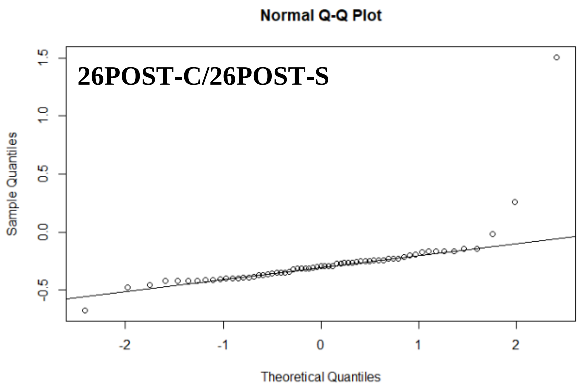

Supplement: Supplementary file 1 [file metabolites-11-00842-s001.zip › supplementary files/Supplementary Information.pdf]
